# Berberine Promotes OATP1B1 Expression and Rosuvastatin Uptake by Inducing Nuclear Translocation of FXR and LXRα

**DOI:** 10.3389/fphar.2020.00375

**Published:** 2020-03-27

**Authors:** Mingyi Liu, Dandan Zhu, Jinhua Wen, Wei Ding, Shibo Huang, Chunhua Xia, Hong Zhang, Yuqing Xiong

**Affiliations:** ^1^Clinical Pharmacology Institute, Nanchang University, Nanchang, China; ^2^Department of Pharmacy, First Affiliated Hospital of Nanchang University, Nanchang, China

**Keywords:** berberine, OATP1B1, FXR, LXRα, rosuvastatin

## Abstract

Berberine, a quinoline alkaloid, can be used in combination with statins to enhance hypolipidemic effects and reduce the dose and side effects of statins. The hypolipidemic effects of statins in the liver are mainly regulated by organic anion transporting polypeptides (OATPs), and the expression of OATPs is regulated by nuclear receptors. Berberine has been reported to affect nuclear receptors. However, whether berberine affects the uptake of statins by regulating nuclear receptor-mediated expression of OATPs remains to be determined. The aim of this study was to investigate the effects of berberine on the expression of OATP1B1 in HepG2 and explore the underlying mechanism. In HepG2 cells, 10–50 μM berberine significantly increased the uptake of rosuvastatin by inducing the expression of OATP1B1 mRNA and protein. Dual-Luciferase reporter assay showed that luciferase activity of hFXR and hLXRα activated OATP1B1 promoter was increased by 2.5–50 μM berberine in a concentration-dependent manner, with half-maximal effective concentration (EC_50_) of 12.19 ± 0.86 and 32.15 ± 2.32 μM, respectively. In addition, after silencing FXR or LXRα by small interfering RNA (siRNA), berberine-induced OATP1B1 expression was significantly attenuated. Western blot analysis of FXR and LXRα protein levels in the cytoplasm and nucleus of HepG2 cells after treatment with berberine showed that berberine induced nuclear translocation and activation of FXR and LXRα. In conclusion, berberine-induced nuclear translocation of FXR and LXRα could activate OATP1B1 promoter, resulting in enhanced expression of OATP1B1 and increased uptake of rosuvastatin.

## Introduction

Statins have been widely used as lipid-lowering drugs because they are inhibitors for hydroxyl methylglutaryl coenzyme A (HMG-CoA) reductase. The combination therapy of statins with bile acid sequestrants, niacin, or ezetimibine have significantly improved efficacy in the treatment of hyperlipidemia (Knapp et al., 2001; Wolfe et al., 2001; Robinson and Davidson, 2006; Huijgen et al., 2010). However, combination therapy have the possibility to increase adverse effects such as muscle toxicity and myopathy, which may be associated with drug interactions mediated by organic anion transporting polypeptide (OATP) transporters (Staffa et al., 2002; Shitara and Sugiyama, 2006; Kitamura et al., 2008; Kunze et al., 2014).

Pregnane X receptor (PXR), constitutive androgen receptor (CAR), farnesoid X receptor (FXR), and liver X receptor α (LXRα) are members of constitutive and ligand-activated nuclear receptor superfamily and play a crucial role in regulating target genes involved in drug metabolism and transport (Urquhart et al., 2007; Staudinger et al., 2013). FXR and LXRα have been characterized as transcriptional factors which regulate the expression of OATP1B1 (Meyer Zu Schwabedissen et al., 2010). Rifampicin significantly increases the expression of OATP1B1 protein and mRNA in hepatocytes by activating PXR (Jigorel et al., 2006).

Berberine is a compound isolated from traditional Chinese medicines and exerts a variety of pharmacological effects such as anti-diabetes, immunoregulation, anti-hypertension, anti-arrhythmia, and lipid-lowering (Lau et al., 2001; Kong et al., 2004; Kong et al., 2009; Gu et al., 2015; Montes et al., 2019; Neag et al., 2018; Belwal et al., 2020;). In addition, berberine regulates metabolic enzymes and transporters to affect the absorption, distribution and metabolism of endogenous and exogenous substances *in vivo*. Berberine increased the blood concentration of cyclosporine A in renal transplanted patients by inhibiting CYP3A4 (Wu et al., 2005). Moreover, berberine can regulate the absorption and metabolism of dextromethorphan, losartan, and midazolam in healthy human by suppressing the activity of CYP2D6, 2C9, and 3A4 (Guo et al., 2012). Berberine-activated LXRα increased the expression of ABCA1 transporter and reduced the accumulation of low-density lipoprotein cholesterol (LDL-c) in macrophages to prevent the formation of foam cells (Lee et al., 2010). Berberine-disrupted STAT5 signaling promoted Ntcp/NTCP expression, resulting in enhanced bile acid uptake (Bu et al., 2017). In addition, berberine-activated RXRα/FXR and RXRα/LXR heterodimers enhanced luciferase activity of FXRE and LXRE (Ruan et al., 2017). Recently, combination of statins and berberine has been clinically used to treat hyperlipidemia. Kong *et al*. demonstrated that combined use of simvastatin and berberine significantly reduced LDL-c levels in rats and hyperlipidemia patients and adverse effects compared to monotherapy (Kong et al., 2008). The combination of berberine and simvastatin remarkably attenuated adverse effects such as rhabdomyolysis and improved the efficacy and safety of treatment (Bei-Bei et al., 2009; Li et al., 2019). Therefore, we speculated that berberine could induce the expression of OATP1B1 transporter through nuclear receptors and boosts the uptake of statins by hepatocytes, thereby improving the lipid-lowering efficacy in combination treatment. Notably, rosuvastatin is not metabolized by CYP450 but is transported by OATP (White, 2002). Therefore, in this study we used rosuvastatin to avoid the interference by CYP450 and used HepG2 cell as the model to investigate the effects of berberine on the expression of OATP1B1 and explore the underlying mechanisms.

## Materials and Methods

### Chemicals

Berberine (purity 98.0%), atorvastatin [internal standard (IS) for rosuvastatin, purity 95.3%], and rosuvastatin (purity 97.6%) were obtained from the National Institutes for Food and Drug Control Products (Beijing, China). GW4064 (purity 99.80%) and GW3965 (purity 99.09%) were purchased from SelleckChem (Houston, TX, USA). Rifampicin (RIF, purity 97.0%) was purchased from Sigma-Aldrich (St. Louis, MO, USA). CITCO (purity 98.0%) was purchased from APExBIO (Beijing, China). FXR, LXRα, OATP1B1, and GAPDH oligonucleotide primers were synthesized by Sangon Biotech Co., Ltd (Shanghai, China). The Dual-Luciferase reporter assay system was purchased from Promega (Madison, WI, USA). Lipofectamine 3000 were purchased from Thermo Fisher Scientific (New York, USA). Rabbit polyclonal antibodies to OATP1B1 (catalog: DF4534), LXRα (catalog: DF6864) and GAPDH (catalog: AF0911) were purchased from Affinity Biosciences (Cincinnati, OH, USA). Rabbit polyclonal antibody to FXR (catalog: ab235094) was purchased from Abcam (Abcam, Cambridge, MA). HANK’s balanced salt mixture (supplemented with Mg^2+^ and Ca^2+^) and 1 mol/L HEPES were supplied by Solarbio (Beijing, China). All other chemicals were of analytical grade and were commercially available. Berberine, GW3964 and GW4064 were all dissolved in dimethylsulfoxide (DMSO) to prepare stock solutions of 10 mM. The working solutions were obtained by diluting the stock solutions with DMEM medium and final concentrations of DMSO were no more than 0.1%.

### Plasmids and Small Interfering RNAs

The pTracer-hFXR, pTracer-hLXRα, and empty pTracer-CMV2 vector were purchased from Maijie Biotech (NanTong, China). The pGL3-OATP1B1 reporter plasmid containing LXRα response element(−128 to +53 bp) and FXR response element (−3040 to −4070 bp) fragment of the SLCO1B1 5′-UTR was constructed by Maijie Biotech (NanTong, China). (Meyer Zu Schwabedissen et al., 2010) The small interfering rna**s (**siRNAs) against hFXR (5′-GAGGAUGCCUCA-GGAAAUA-3′) and hLXRα (5′-AACTCAATGATGCTGAGTT-3′) and negative control scramble siRNA were purchased from Maijie Biotech (NanTong, China).

### Cell Culture

The human liver carcinoma cell line HepG2 was provided by Novo Biotechnology (Shanghai, China) and cultured in Dulbecco’s modified Eagle medium (DMEM, Solarbio Co., Ltd, Beijing, China) supplemented with 10% fetal bovine serum (FBS, Biological Industries, Israel) as described previously (Zhong et al., 2018). Cells were cultured to 70%–80% confluency and then treated with the chemicals for 24 h, and cells treated with 0.1% DMSO (generally considered noncytotoxic) were used as the blank control.

### Real-Time PCR

Total RNA was isolated from HepG2 cells using EasySpin cell RNA extraction kit (Aidlab Biotechnologies Co., Ltd, Beijing, China) following the manufacturer’s instruction. RNA (1.5 μg) was first reverse-transcribed into cDNA using Transcriptor First-strand cDNA Synthesis Kit (TransGen Biotech. Beijing, China), and real-time PCR was performed using Premix Ex Taq™ Probe qPCR (TaKaRa Biotech, Kyoto, Japan) following the manufacturer’s instructions. The following primers were used: OATP1B1, 5′- ACCTGCTAGA CAGGGTGAGAT-3′ (forward) and 5′- ACCTGCTAGACAGGG-TGAGAT-3′ (reverse); FXR, 5′- TCAGCCAAC ATTCCCATC-3′ (forward) and 5′- CCTGTGACAAAGAAGCCG-3′ (reverse); LXRα, 5′-CCACTGCCCCATGGACA-CCT-3′ (forward) and 5′- TGTTCCTCCTCT TGCCGCTTC-3′ (reverse); GAPDH, 5′- CAGGGCTGCTTTTAACTCTGGT-3′ (forward) and 5′- GATTTTGGAGGGA-TCTCGCT-3′ (reverse). The data were calculated according to the comparative △△CT method and presented as relative fold of the control.

### Western Blot Analysis

Cells were lysed with RIPA buffer (Applygen Gene Technology Co., Ltd. Beijing, China), and the nuclear and cytoplasmic proteins were separated and extracted using a nuclear and cytoplasmic extraction kit (Boster, Wuhan, China) according to the manufacturer’s instructions. Protein concentrations were quantified with BCA protein assay kit (Vazyme Biotech, Nanjing, China). Proteins (20 μg/sample) were separated using 10% SDS–PAGE and transferred onto polyvinylidene fluoride (PVDF) membranes. Subsequently, the membranes were blocked for 2 h with 5% skim milk and then incubated overnight at 4°C with primary antibodies. The membranes were washed in TBS and then incubated with horseradish peroxidase-conjugated anti-rabbit or anti-rat IgG antibody (Santa Cruz, CA, USA) for 1 h at room temperature. GAPDH and Lamin B1 were used as loading controls. The bands were detected using a Bio-Rad ChemiDoc XRS imaging system (Bio-Rad Laboratories).

### Rosuvastatin Uptake Assay

Rosuvastatin uptake assay in HepG 2 cells was performed as previously described (Li et al., 2012). Briefly, the cells were seeded at 2 × 10^5^/well into 24-well plates and cultured for 24 h, and then treated with a medium containing berberine or a blank control (0.1% DMSO) at 37°C for 24 h. In the uptake experiments, cells were washed three times with HBS–HEPES (99:1) uptake buffer at 37°C, and then the cells were incubated for 10 min in uptake buffer containing 20 μM rosuvastatin. After the incubation, the buffer was quickly aspirated, the cells were washed three times with ice-cold HBSS–HEPES buffer, and repeatedly thawed three times at −80°C and room temperature. Finally, 100 μl cell lysate was spiked with 20 μl IS (10 ng/ml atorvastatin), and 200 μl methanol was added. The mixture was then vortexed for 1 min and centrifuged at 10,000*g* for 10 min, with an aliquot (10 µl) automatically injected into the LC-MS/MS system for analysis, and protein content was determined by BCA method. Three independent experiments were performed in triplicates.

### Quantification of Rosuvastatin by LC-MS/MS

The concentration of rosuvastatin in cells was determined by LC-MS/MS system consisted of Shimadzu LC-20AB pumps (Shimadzu Corporation, Kyoto, Japan) and an AB SCIEX API 4000 mass spectrometer (Applied Biosystems/SCIEX, Foster, CA, USA). Data acquisition was performed using Analyst 1.6.1 software (AB SCIEX). Chromatographic separation was achieved on a Luna C18 column (50 × 2.0 mm i.d., 5 µm; Phenomenex Technologies). The mobile phase consisted of 10-mM ammonium formate (A) and acetonitrile (B) using a gradient elution of 40-90% B at 0.0–1.0 min, 90%–90% B at 1.0–2.5 min, and 40%–40% B at 2.51–3.5 min. The flow rate was 0.4 ml/min, the operating temperature was 25°C.

Samples were ionized utilizing an electrospray-ionization probe in the positive-ion mode, and quantification was performed using the multiple-reaction monitoring (MRM) method, with the precursor-to-product transition being m/z 482.3→258.2 for rosuvastatin and m/z 559.2→440.0 for atorvastatin (IS). Nitrogen was used as the curtain and auxiliary gas, and air was used as the nebulizer gas under the following conditions: curtain gas, 40 psi; ion-spray voltage, 5500 V; nebulizer gas, 50 psi; auxiliary gas, 50 psi; and turbo temperature, 500°C. The collision energy (CE) was 45 V for rosuvastatin and 28V for atorvastatin, and the declustering potential (DP) was 118 V for rosuvastatin and 100 V for atorvastatin.

### Dual Luciferase Assay

pTracer-hFXR, pTracer-hLXRα, and empty pTracer-CMV2 vector were purchased from Maijie Biotech (NanTong, China). The pGL3-OATP1B1 vector was prepared as described (Meyer Zu Schwabedissen et al., 2010) containing LXRα response element (−128 to +53 bp) and FXR response element (−3,040 to −4,070 bp) fragment of the *SLCO1B1* 5′-UTR, and empty plasmid pGL3-Basic, internal reference Renilla luciferase plasmid pRL-TK were purchased from Maijie Biotech. Corresponding plasmids were transfected into HepG2 cells with Lipofectamine 3000 transfection reagent following the manufacturer’s instructions. Finally, the cells were harvested and cell lysates were assayed for firefly activities normalized against the activities of co-transfected renilla luciferase using a dual-luciferase kit (Promega).

### RNA Interference

The siRNA against hFXR or hLXRα and negative control scramble siRNA were purchased from Maijie Biotech (NanTong, China). siFXR (5′-GAGGAUGCCUCA-GGAAAUA-3′) or siLXRα (5′-AACTCAATGATGCTGAGTT-3′) was transfected into HepG2 cells at the final concentration of 50 nmol/L. The knockdown efficiency was detected by Western blot analysis.

### Statistical Analysis

The data from three independent experiment were presented as mean ± standard deviation (mean ± SD), and one-way ANOVA was used to determine the differences among the groups using GraphPad Prism 5.0. p < 0.05 indicated that the differences were significant.

## Results

### Effect of Berberine on OATP1B1 Expression in HepG2 Cells

**Figure 1:**
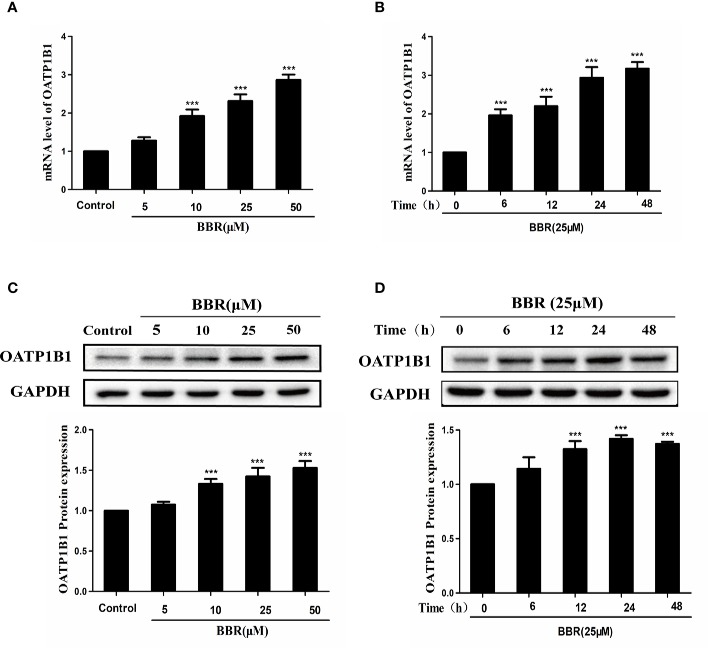
Effects of berberine on OATP1B1 expression in HepG2 cells. **(A, B)**. *OATP1B1* mRNA expression was analyzed by real-time PCR in HepG2 cells after treatment with increasing concentrations of berberine (5, 10, 25, and 50 μM) for 24 h or treatment with 25 μM berberine at a series of time points (6, 12, 24, and 48 h). **(C, D)** OATP1B1 protein levels were determined by Western blotting in HepG2 cells after treatment with increasing concentrations of berberine (5, 10, 25, and 50 μM) for 24 h or treatment with 25 μM berberine at a series of time points (6, 12, 24, and 48 h). Data are represent as mean ± SD from triplicate independent experiments after being normalized to GAPDH, and 0.1% dimethylsulfoxide (DMSO) were used as the negative control. ***P < 0.001 compared to the control.

To investigate the effects of berberine on the expression of OATP1B1, HepG2 cells were treated with a series of concentrations of berberine (5, 10, 25, and 50 μM) for 24 h, or treated with 25 μM berberine for a series of time (6, 12, 24, and 48 h). Real-time PCR showed that berberine significantly upregulated OATP1B1 mRNA levels in a concentration and time- dependent manner (**Figures 1A, B**). Western blot analysis showed that 10–50 μM berberine enhanced the expression of OATP1B1 protein in a concentration-dependent manner after 24-h treatment (**Figures 1C, D**).

### Berberine Increased Rosuvastatin Uptake in HepG 2 Cells

**Figure 2:**
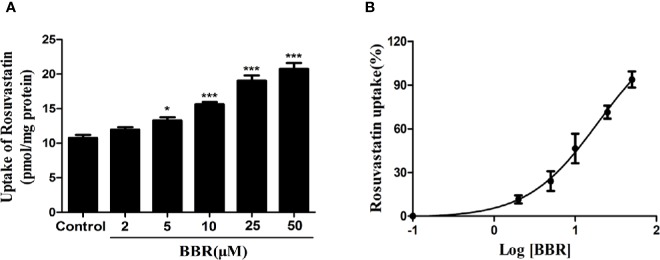
Berberine enhanced rosuvastatin uptake in HepG2 cells. **(A, B)** Rosuvastatin uptake assay was conducted in HepG2 cells treated with increasing concentrations (2, 5, 10, 25, and 50 μM) of berberine for 24 h. The concentrations of rosuvastatin were determined by HPLC-MS/MS. DMSO (0.1%) was used as the negative control. Data are expressed as mean ± SD of triplicate independent experiments,*P < 0.05, ***P < 0.001 compared to the control.

To investigate the effects of berberine on OATP1B1 transport function, the time- and concentration-dependent uptake assays were conducted, and uptake kinetic parameters were examined in a series concentration or time point (data not shown). The HPLC-MS-MS method for the determination of rosuvastatin was validated with selectivity, precision, accuracy, extract recovery, and matrix effect (**Supplementary Figure 1**). The uptake of rosuvastatin was linear with time over the first 10 min, and the *Km* (Michaelis constant) was determined to be 21.50 ± 1.77 μM, thus we performed the specific rosuvastatin uptake test under the condition of 20 μM and 10 min. After treatment with increasing concentrations (2, 5, 10, 25, and 50 μM) of berberine for 24 h, the uptake of rosuvastatin was increased 1.24-fold (5 μM berberine-treated), 1.42-fold (10 μM berberine-treated), 1.78-fold (25 μM berberine-treated), and 1.93-fold (50 μM berberine-treated) compared to control, respectively (**Figure 2A**). The half-maximal effective concentration (EC_50_) value was measured to be 19.01 ± 1.21 μM (**Figure 2B**).

### Berberine Enhanced FXR and LXRα Mediated Activation of OATP1B1 Promoter

**Figure 3:**
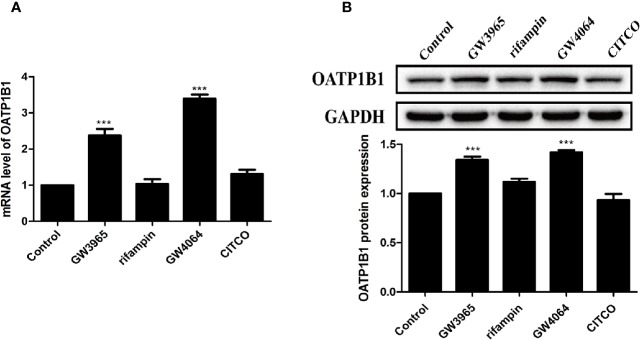
Effects of diverse nuclear receptors activators on OATP1B1 expression in HepG2 cells. HepG2 cells were treated with 10 μM GW3965, 10 μM rifampin, 10 μM GW4064, or 10 μM CITCO for 24 h, OATP1B1 expression was determined by real-time PCR **(A)** and Western blot analysis **(B)**. DMSO (0.1%) was used as the negative control. Data are expressed as mean ± SD from triplicate independent experiments, ***P < 0.001 compared to the control.

HepG2 cells were treated with 10 μM of nuclear receptor ligands including rifampicin (PXR ligand), CITCO (CAR ligand), GW4064 (FXR ligand), or GW3965 (LXRα ligand) for 24 h. PCR and Western blot analysis showed that only GW3965 (a specific LXRα agonist) and GW4064 (a specific FXR agonist) markedly upregulated the expression of OATP1B1 mRNA and protein (**Figures 3A, B**).

**Figure 4:**
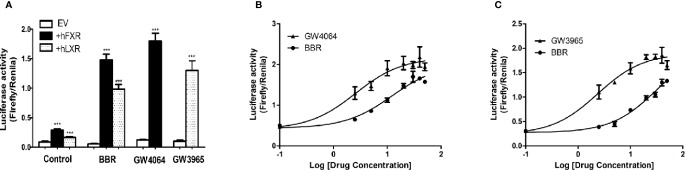
Effect of berberine on OATP1B1 promoter in HepG2 cells transiently transfected with hFXR or hLXRα. HepG2 cells transiently co-transfected with pTracer-hFXR or pTracer-hLXRα and reporter vector pGL3-OATP1B1 were treated with berberine (25 μM), GW4064 (10 μM), or GW3965 (10 μM) **(A)**, and increasing concentrations of berberine (2.5, 5, 10, 20, 30, 40, 50 μM), GW4064 (2.5, 5, 10, 20, 30, 40, 50 μM), or GW3964 (2.5, 5, 10, 20, 30, 40, 50 μM) **(B, C)**. The fold induction of luciferase activity was determined using a Dual-Luciferase reporter assay system. DMSO (0.1%) was used as the negative control. Data are expressed as mean ± SD of triplicate independent experiments, ***P < 0.001 compared to the control.

Next we performed dual luciferase reporter assay to investigate the potency of berberine on activating transcriptional activity of LXRα and FXR on *OATP1B1* promoter. As shown in **Figure 4A**, luciferase activity significantly increased after treatment with 25 μM berberine or/and 10 μM corresponding ligands. Meanwhile, the EC_50_ value was 12.19 ± 0.86 μM in HepG2-hFXR-OATP1B1-luc cells (**Figure 4B**), and the EC_50_ value was 32.15 ± 2.32 μM in HepG2-hLXRα-OATP1B1-luc cells (**Figure 4C**), while the EC_50_ in the two cell lines were 2.56 ± 0.21 and 2.37 ± 0.36 μM, respectively, after GW4064 and GW3965 treatment. These data suggest that berberine could improve hFXR or hLXRα-mediated activation of OATP1B1 luciferase activity in a concentration-dependent manner,

### Berberine Enhanced FXR and LXRα Induced Expression of OATP1B1 Protein and Transport Function

**Figure 5:**
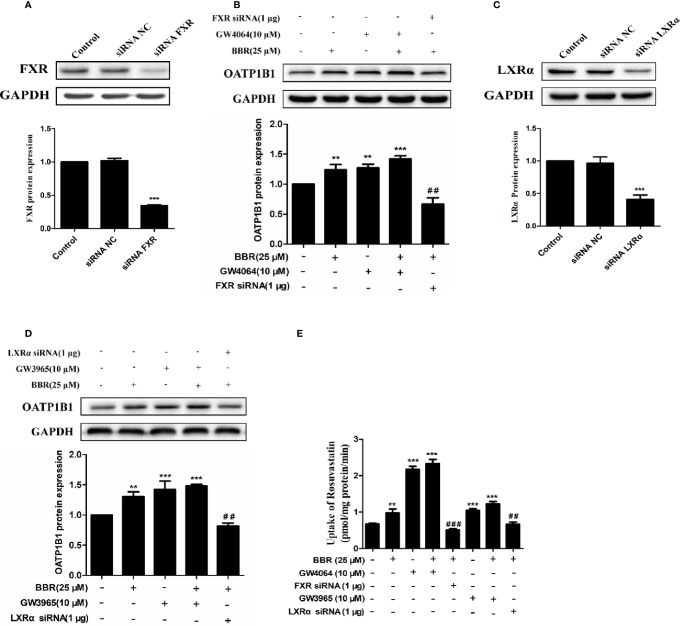
Silencing *hFXR* and *hLXRα* expression abolished berberine-induced OATP1B1 expression in HepG2 cells. HepG2 cells were transiently transfected with small interfering RNA (siRNA)-hFXR or siRNA-hLXRα, and 48 h later protein levels of FXR **(A)** and LXRα **(C)** were examined by Western blot. HepG2 cells were then treated with berberine (25 μM), GW4064, or GW3965 (10 μM) for 24 h, OATP1B1 protein levels were analyzed by Western blot **(B, D)**. **(E)** Rosuvastatin uptake assay was conducted in HepG2 cells treated with berberine (25 μM), GW4064 or GW3965 (10 μM), or FXR siRNA or LXRα siRNA for 24 h. The concentrations of rosuvastatin were determined by HPLC-MS/MS. DMSO (0.1%) was used as negative control. Data are expressed as mean ± SD of triplicate independent experiments, **P < 0.01, ***P < 0.001 compared to the control. ^##^P < 0.01 and ^###^P < 0.001 compared to 25 μM BBR group.

To further investigate the potency of LXRα and FXR activated by berberine on regulating OATP1B1 expression, HepG2 cell models with silenced or induced FXR and LXRα activities were constructed. First, Western blot analysis confirmed that transfection of siRNA-hFXR or siRNA-hLXRα into HepG2 cells significantly reduced FXR and LXRα protein levels compared to control group (**Figures 5A, C**). Subsequently, HepG2 cells were treated with berberine, GW4064, GW3965, or/and FXR/LXRα siRNAs. As shown in **Figures 5B, D**, the induction of OATP1B1 expression by GW4064 and GW3965 was significantly increased by berberine, while FXR or LXRα siRNA eliminated the upregulation of OATP1B1 by berberine. Furthermore, berberine, GW4064 and GW3965 significantly increased the uptake of rosuvastatin by OATP1B1, but knockdown of FXR or LXRα significantly reduced berberine stimulated rosuvastatin uptake by OATP1B1 (**Figure 5E**). These results indicate that FXR and LXRα participate in the upregulation of OATP1B1 expression by berberine.

### Berberine Induced Nuclear Translocation of FXR and LXRα

**Figure 6:**
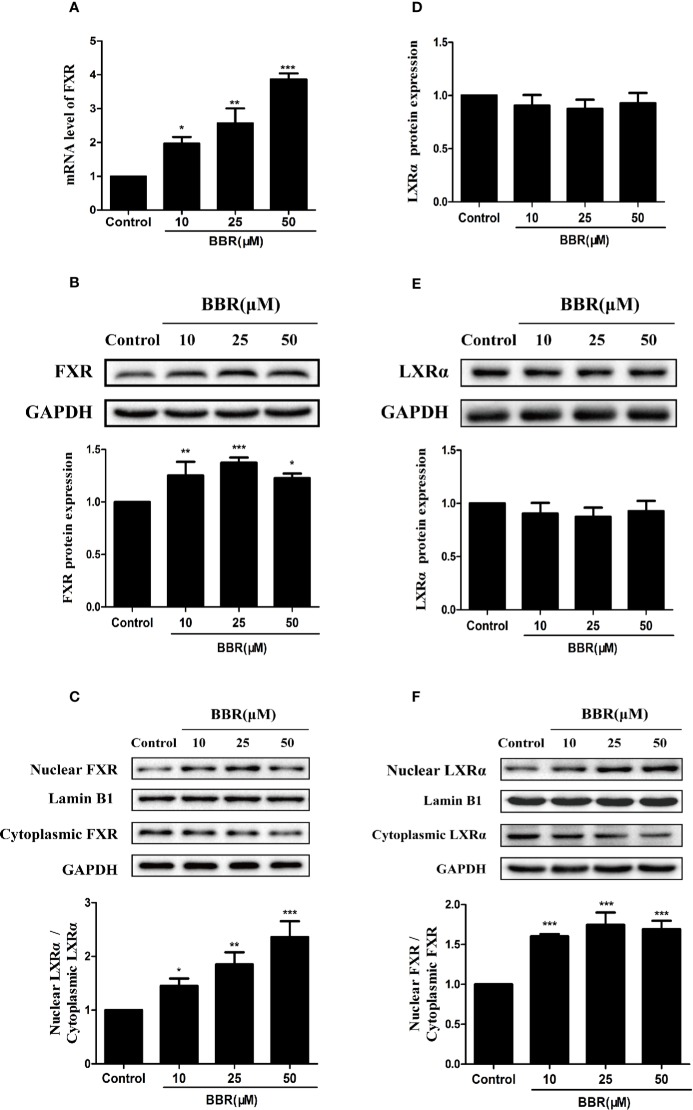
Effects of berberine on FXR and LXRα expression and distribution in HepG2 cells. HepG2 cells were treated with increasing concentrations of berberine (10, 25, and 50 μM) for 24 h. FXR **(A)** and LXRα **(D)** mRNA levels were measured by PCR; protein levels were measured by Western blot **(B, E)**. The nuclear and cytoplasmic FXR **(C)** and LXRα **(F)** protein levels were quantified by Western blot. The determination of mRNA and protein were normalized to GAPDH, nuclear and cytoplasmic protein was normalized to Lamin B1 and GAPDH, respectively. DMSO (0.1%) was used as the negative control. Data are expressed as mean ± SD of triplicate independent experiments,*P < 0.05, **P < 0.01, ***P < 0.001 compared to the control.

Previous studies reported that nuclear receptors such as FXR and LXRα regulate the expression of target genes after ligand-induced nuclear translocation (Lee et al., 2010; Xu et al., 2016). Therefore, we investigated the effect of berberine on the expression and distribution of FXR and LXRα in HepG2 cells. PCR analysis showed that berberine significantly increased FXR mRNA expression in a concentration-dependent manner (**Figure 6A**). Consistently, Western blot analysis showed that berberine upregulated the expression of FXR protein in HepG2 cells, except that the induction of FXR protein by berberine was attenuated slightly at the highest dose of 50 μM (**Figure 6B**). However, berberine had no significantly effect on the expression of LXRα at both mRNA and protein levels (**Figures 6D, E**). Subsequently, cytoplasmic proteins and nuclear proteins were isolated and Western blot analysis showed that nuclear FXR and LXRα protein levels increased significantly after treatment with berberine for 24 h, while cytoplasmic FXR and LXRα protein levels decreased slightly (**Figures 6C, F**). These results indicate that berberine could promote nuclear translocation of FXR and LXRα.

## Discussion

In this study, we provide the first evidence that berberine enhanced FXR and LXRα mediated upregulation of OATP1B1 expression, resulting in enhanced uptake of the substrate rosuvastatin in HepG2 cells, which may be responsible for improved lipid-lowering efficacy in combination with statins.

Berberine is a new type of hypolipidemic drug widely used with favorable clinical efficacy and safety (Kong et al., 2004; Kong et al., 2009). Studies have reported that the combination of berberine with simvastatin can significantly enhance the hypolipidemic efficacy in patients and rats with hyperlipidemia, reduce the dose of statins, and lower the risk of adverse reactions (Bei-Bei et al., 2009; Kong et al., 2009; Li et al., 2019). Notably, we found that rosuvastatin had rapid uptake in HepG2 cells, this may be explained by that rosuvastatin is mainly transported by OATP1B1 and OATP1B3, which are highly expressed in the liver (Hagenbuch and Meier, 2003) Furthermore, we showed that berberine upregulated the expression of OATP1B1 transporter at both mRNA and protein levels in HepG2 cells, which could promote rosuvastatin uptake in HepG2 cells.

FXR and LXRα are widely involved in the regulation of transporters expression *in vivo*. Ligand-activated FXR can upregulate BSEP transcription, inhibit NTCP transcription or induce PLTP and CYP7A1 expression to promote bile acid secretion, suppress uptake of bile acid in the liver or increase cholesterol metabolism (Mak et al., 2002). LXRα regulates the metabolism of bile acids and cholesterol in the liver by regulating target genes such as CYP7, ABC transporter ABCA1, and lipoprotein lipase (Peet et al., 1998; Schmitz and Langmann, 2001). It has been reported that nuclear receptors FXR and LXRα can act on OATP1B1 promoter to regulate the expression (Meyer Zu Schwabedissen et al., 2010). Our study demonstrated that berberine regulated the expression of OATP1B1 by activating FXR and LXRα. This finding was confirmed by Dual-Luciferase reporter assay. The results indicated that berberine had stronger induction on hFXR-mediated transcriptional activation of OATP1B1 than on hLXRα, but the effect was weaker than classical agonists GW4064 and GW3965. However, the combination of berberine and the corresponding agonists can further improve the expression of target proteins. Silencing FXR or LXRα by siRNA dramatically diminished the upregulation of OATP1B1expression by berberine. These results confirmed that FXR and LXRα mediate the effects of berberine on the upregulation of OATP1B1 expression.

However, the underlying mechanism by which berberine activates FXR and LXRα in HepG2 cells is unclear. LXRα and FXR have been identified as critical nuclear receptors which bind to the promoters of target genes to regulate transcriptional activity of downstream target genes after ligand-activated nuclear translocation (Meyer Zu Schwabedissen et al., 2010; Xu et al., 2016; Zhou et al., 2016). In this study we found that berberine promoted nuclear translocation and activation of FXR and LXRα, similar to other Chinese herbal medicines such as ginkgolide b (Zhou et al., 2016) and dihydroartemisinin (Xu et al., 2016). In addition, berberine significantly increased the expression of FXR protein and mRNA at 10 and 25 μM, but only moderately upregulated the expression of FXR protein at 50 μM, while the mRNA expression was still significantly upregulated, suggesting that FXR may be subjected to a series of post-transcriptional regulations, such as phosphorylation, acetylation, and glycosylation (Chang, 2009; Sugatani et al., 2014).

Although statins are known as HMG-CoA reductase inhibitors, previous study suggested that berberine inhibited HMG-CoA reductase activity *via* increased phosphorylation of HMG-CoA reductase, leading to reduced hepatic cholesterol level (Wu et al., 2011). In addition, recent reports showed that berberine could inhibit lipogenesis by targeting sterol regulatory element-binding protein (SREBP) related signaling (Yunxin et al., 2019; Zhu et al., 2019). Therefore, furthers studies are needed to demonstrate that berberine and statins in combination can inhibit SREBP signaling and HMG-Co reductase activity to achieve enhanced hypolipidemic effects.

In summary, our results suggest that berberine upregulates the expression of OATP1B1 in HepG2 cells by inducing nuclear translocation of FXR and LXRα, which then activate the expression of OATP1B1 and increase the uptake of rosuvastatin.

## Data Availability Statement

All datasets generated for this study are included in the article/**Supplementary Material**.

## Author Contributions

YX, HZ, and CX participated in study design. ML, DZ, and WD conducted the experiments and analyzed the data. JW and SH contributed to the writing of the manuscript.

## Funding

This work was supported by the Natural Science Foundation of China (No. 81673506).

## Conflict of Interest

The authors declare that the research was conducted in the absence of any commercial or financial relationships that could be construed as a potential conflict of interest.
