# Supplementary material for: Berberine Promotes OATP1B1 Expression and Rosuvastatin Uptake by Inducing Nuclear Translocation of FXR and LXRα
Source: Front Pharmacol. 2020 Mar 27;11:375. doi: 10.3389/fphar.2020.00375 (PMC7118773; doi:10.3389/fphar.2020.00375)
Supplement: Supplementary file 1 [file Image_1.pdf]

A

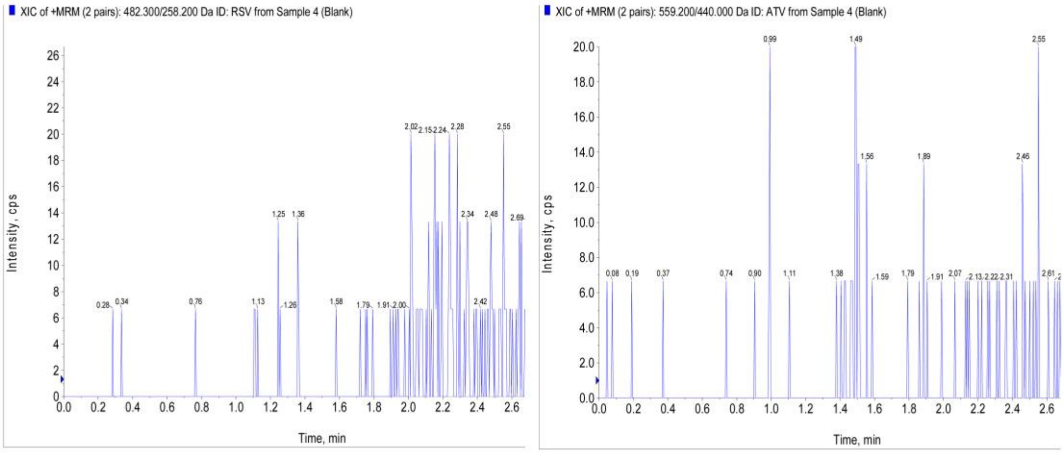
XIC of +MRM (2 pairs): 482.300/258.200 Da ID: RSV from Sample 4 (Blank)
Intensity, cps
Time, min
XIC of +MRM (2 pairs): 559.200/440.000 Da ID: ATV from Sample 4 (Blank)
Intensity, cps
Time, min

B

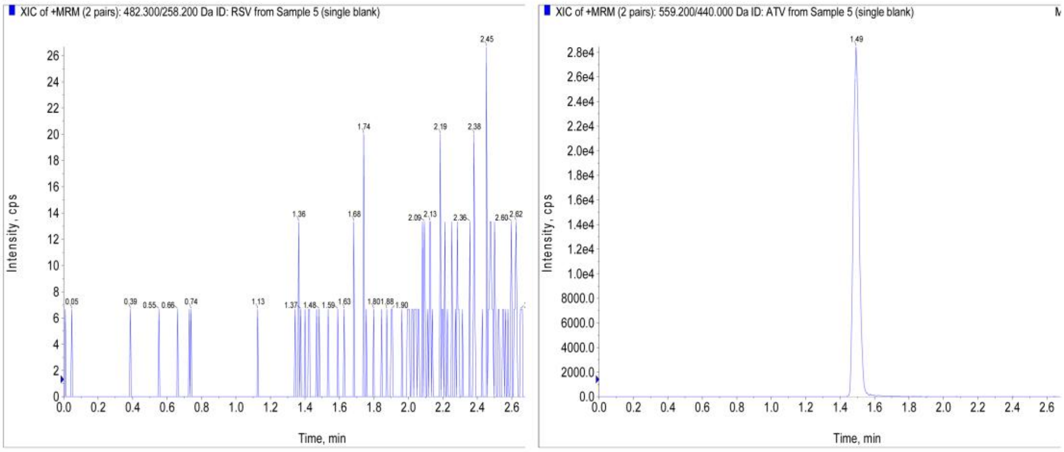
XIC of +MRM (2 pairs): 482.300/258.200 Da ID: RSV from Sample 5 (single blank)
Intensity, cps
Time, min
XIC of +MRM (2 pairs): 559.200/440.000 Da ID: ATV from Sample 5 (single blank)
Intensity, cps
Time, min

C

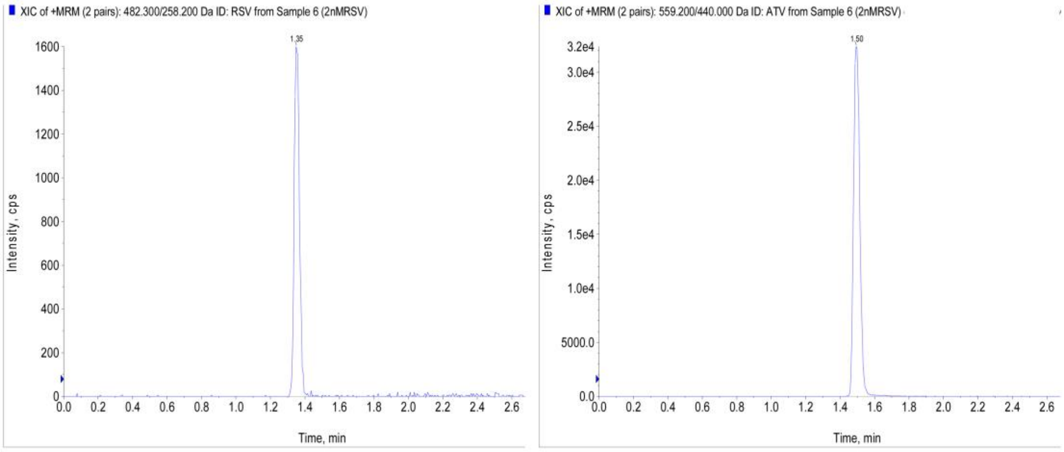
XIC of +MRM (2 pairs): 482.300/258.200 Da ID: RSV from Sample 6 (2nMRSV)
Intensity, cps
Time, min
XIC of +MRM (2 pairs): 559.200/440.000 Da ID: ATV from Sample 6 (2nMRSV)
Intensity, cps
Time, min

Supplemental figure 1. Chromatograms of rosuvastatin (RSV) and atorvastatin (ATV) in HepG2 cell lysate: A, Blank sample; B, Blank sample with ATV; C, Cell lysate spiked with LLOQ at concentration of 2.00 nM. ATV was used as internal standard.
